# Oxoisoaporphine as Potent Telomerase Inhibitor

**DOI:** 10.3390/molecules21111534

**Published:** 2016-11-14

**Authors:** Zu-Zhuang Wei, Qi-Pin Qin, Jia-Nian Chen, Zhen-Feng Chen

**Affiliations:** State Key Laboratory for Chemistry and Molecular Engineering of Medicinal Resources, School of Chemistry and Pharmacy, Guangxi Normal University, 15 Yucai Road, Guilin 541004, China; weizuzhuang@126.com (Z.-Z.W.); qinqipin2007@126.com (Q.-P.Q.); cjn288@sina.com (J.-N.C.)

**Keywords:** oxoisoaporphine, telomerase, molecular docking

## Abstract

Two compounds previously isolated from traditional Chinese medicine, *Menispermum dauricum* (DC), 6-hydroxyl-oxoisoaporphine (H-L^a^), and 4,6-di(2-pyridinyl)benzo[*h*]isoindolo[4,5,6-*de*]quinolin-8(5*H*)-one (H-L^b^), were known to have in vitro antitumor activity and to selectively bind human telomeric, c-myc, and bcl-2 G-quadruplexes (G4s). In this study, the binding properties of these two compounds to telomerase were investigated through molecular docking and telomeric repeat amplication protocol and silver staining assay (TRAP-silver staining assay). The binding energies bound to human telomerase RNA were calculated by molecular docking to be −6.43 and −9.76 kcal/mol for H-L^a^ and H-L^b^, respectively. Compared with H-L^a^, the ligand H-L^b^ more strongly inhibited telomerase activity in the SK-OV-3 cells model.

## 1. Introduction

Previous studies found that telomerase enzyme complexes or telomerase inhibitors play a key role in tumorigenesis and telomere maintenance, and suggested that these structures may be an entry point for devising novel anticancer drugs [1,2,3]. Various strategies have been proposed to inhibit telomerase in cellular immortalization. A promising method among them aims to disrupt the telomere–telomerase interaction with small molecules as interfering ligands. The ligand binds the enzyme substrate to prevent telomere elongation or formation of the telomere–telomerase complex [4,5]. In addition, a large number of G4s structural studies have shown that telomeric DNA terminates in a single-stranded 3′ over hang which can form higher-order conformations termed G4s, such as telomeric, bcl-2, and c-myc G-quadruplexes (G4s) [6,7,8,9]. Therefore, small molecules that selectively bind and stabilize these structures (e.g., telomeric G4 and other G4s DNA) can influence telomere maintenance and potentially serve as therapeutic agents [1,2,3,4,5,6,7,8,9].

In addition, it has been reported that the identification of proteins in eukaryotic cells that recognize, generate, or alter G4s in vitro with remarkable specificity suggests that these structures were formed in vivo [10,11]. Because human tumor cells and numerous neoplasia have been found to be accompanied with aberrant telomere length regulation, potent G-quadruplexes (G4 DNAs) with high selectivity against human cancer cells and low toxicity against normal cells are considered as new molecular target in cancer therapeutics [12]. Furthermore, the formation of G4 (G-quadruplex) by telomeric DNA inhibits the activity of telomerase, an enzyme not found in most normal somatic cells, but present in 85%–90% of cancer/tumor cells and contributing to the immortality of these cells [12]. It has been hypothesized that telomerase has to be reactivated in order to maintain telomere length, and consequently allow tumor cells to undergo sustained proliferation. Hence, novel G4s ligands that can target telomerase are deemed to be promising therapeutic agents [13,14,15].

**Figure 1:**
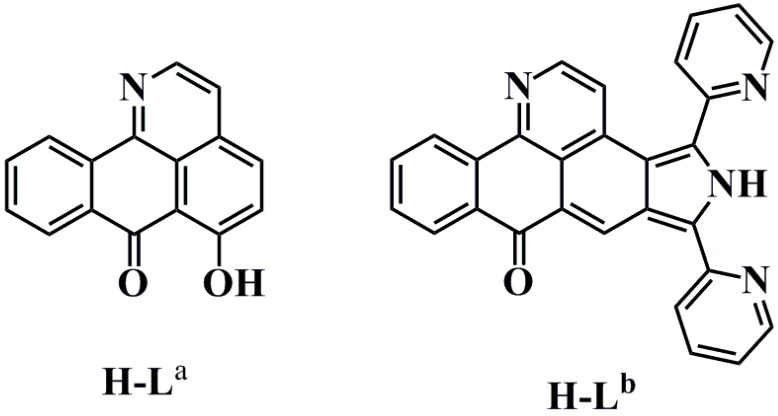
Chemical structure of oxoisoaporphine ligands H-L^a^ (6-hydroxyl-oxoisoaporphine) and H-L^b^ (6-di(2-pyridinyl)benzo[*h*]isoindolo[4,5,6-*de*]quinolin-8(5*H*)-one).

Natural and synthetic alkaloids (including liriodenine, formamide oxoaporphine, oxoisoaporphine, and its derivatives, etc.) and their complexes are important molecules that bind G4s (such as bcl-2, telomere, and/or c-myc G4s) [16]. A small number of formamide oxoaporphine and oxoisoaporphine metal complexes are reported to be G-quadruplex ligands or telomerase inhibitors [17,18,19,20,21]. In this paper, we use molecular docking and a TRAP-silver staining assay (telomeric repeat amplication protocol and silver staining assay) to investigate the binding properties of two oxoisoaporphine ligands (6-hydroxyl-oxoisoaporphine, H-L^a^ [19]; and 6-di(2-pyridinyl)benzo[*h*]isoindolo[4,5,6-*de*]quinolin-8(5*H*)-one, H-L^b^ [21]) to telomerase (Figure 1).

## 2. Results and Discussion

### 2.1. Molecular Docking

**Figure 2:**
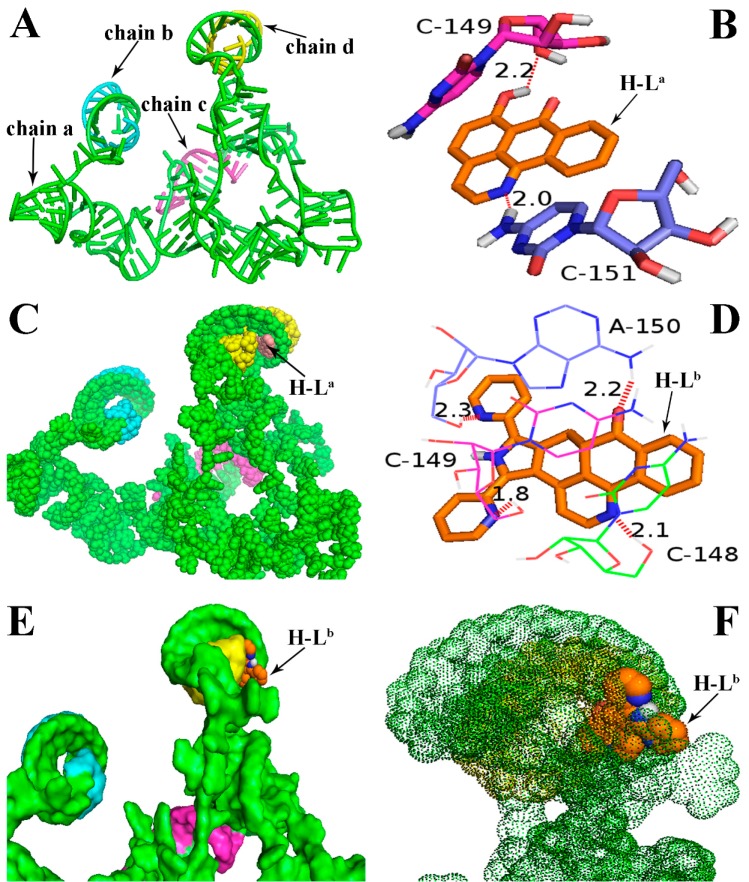
Binding mode of the ligands H-L^a^ and H-L^b^ with human telomerase RNA (hTR). (**A**) Three-dimensional conformation model of hTR (PDB ID: 2INA), which was not reported in the RCSB protein data bank (http://www.rcsb.org). The bases and chains are represented as a cartoon; (**B**) Two H-bonds were formed between H-L^a^ and the receptor 2INA; (**C**) H-L^a^ was embedded in the active pocket; (**D**) Four H-bonds were formed between H-L^b^ and the receptor 2INA; (**E**,**F**) H-L^b^ was surrounded by a “fog” formed by chains a and d. In Figure 2B,D, the ligands are represented as ball-and-stick models and colored by atom type. Hydrogen bonds are represented by red dotted lines. White: hydrogen atom; red: oxygen atom; dark blue: nitrogen atom; orange: the backbone and carbon atom of the ligands H-L^a^ and H-L^b^.

Molecular docking analyses were performed using the AutoDock software (Version: 4.2.6, The Scripps Research Institute, La Jolla, CA, USA) in order to investigate the probable binding mode between the ligand (H-L^a^ and H-L^b^) and human telomerase. Human telomerase is the enzyme that maintains the length of telomeres, which mainly consists of three components: a core reverse transcriptase protein (hTERT), telomerase RNA (hTR), and several species-specific proteins [22]. Despite its significance as an almost universal cancer target, the understanding of human telomerase and the development of specific inhibitors have been hampered by the limited data about this enzyme’s three-dimensional structure. To date, the crystal structures of hTERT and hTR have not been reported, though other species’ telomerases—such as the telomerases of *Tetrahymena thermophila* and *Tribolium castaneum*—have already been decoded several years before [23,24,25]. Therefore, in many reports, the molecular docking analyses of the inhibitors is mainly based on non-human species’ telomerases [25], though as we all know, the human telomerase structure is very different from that of other species. In this study, with the aid of the receptor model (PDB code: 2INA) which has been reported previously [22,26], the above two ligands were successfully docked into the hTR pocket for the first time (Figure 2). As shown in Figure 2A, hTR consists of four intertwined chains: a (in green), b (in blue), c (in red), and d (in yellow). The ligand H-L^a^ was mainly interacted with the chains a and d, and two H-bonds were formed. The first H-bond with a distance of 2.0 Å formed between 7-carbonyl oxygen atom of H-L^a^ and a hydrogen atom in the base moiety C-151 of chain a (in Figure 2B, “C-151” refers to NO. 151 cytidine). The second H-bond with a distance of 2.2 Å formed between one 6-hydroxy group (OH) of the ligand and the oxygen atom in the nucleoside moiety (OH···O) of chain a. Chains a and d were helically wound and formed a larger pocket, in which the ligand H-L^a^ was easily embedded (Figure 2C). Very different to the ligand H-L^a^, the first two H-bonds were formed between two nitrogen atoms of H-L^b^ and cytidine bases (C-148 and C-149), the third H-bond with a distance of 2.3 Å was formed between the nitrogen atom in another pyridine unit of H-L^b^ and adenosine base (A-150); simultaneously, the sole carbonyl oxygen atom of H-L^b^ formed the fourth H-bond (CO···HN) with a distance of 2.2 Å (Figure 2D). As described above, because the binding pocket could hold those molecules with larger volume, the ligand H-L^b^ could be almost perfectly embedded in this active pocket (Figure 2E). The binding energies bound to hTR were calculated to be −6.43 and −9.76 kcal/mol for H-L^a^ and H-L^b^, respectively. In the binding model, the ligand H-L^b^ was surrounded by a thick “fog” formed by chains a and d (Figure 2F). In our opinion, compared with H-L^a^, besides an additional two H-bonds, the more suitable molecular volume of H-L^b^ resulted in stronger affinity to hTR. The molecular docking results further supported the previous hypothesis that H-L^b^ was more cytotoxic and could more strongly inhibit telomerase.

### 2.2. In Vitro Cytotoxicity

**Figure 3:**
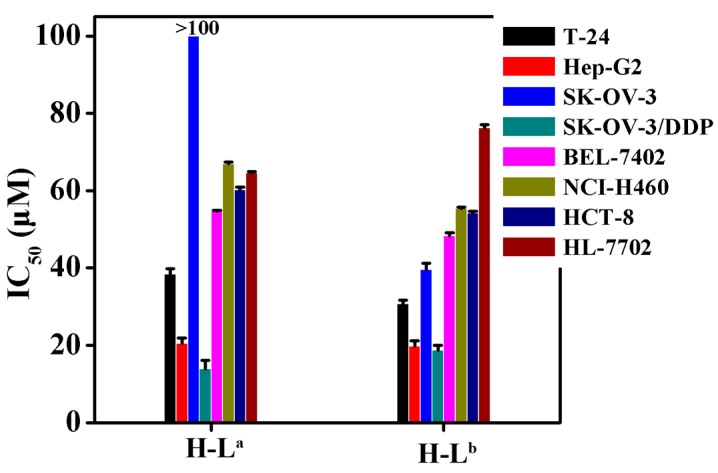
IC_50_ values (µM) of H-L^a^ and H-L^b^ for selected cells (T-24, HCT-8, Hep-G2, SK-OV-3/DDP, BEL-7402, HL-7702, SK-OV-3 and NCI-H460 human cell lines).

The IC_50_ values of the two oxoisoaporphine ligands H-L^a^ and H-L^b^ were measured using the 3-(4,5-dimethylthiazol-2-yl)-2,5-diphenyltetrazolium bromide (MTT) method against the human normal liver HL-7702 cell line [19,21] and several human cancer cell lines (including T-24, HCT-8, Hep-G2, SK-OV-3/DDP, SK-OV-3, BEL-7402, and NCI-H460 tumor cells). Figure 3 shows that, compared with H-L^a^, H-L^b^ exhibited stronger cytotoxicity against all tested cell lines except SK-OV-3/DDP. Notably, H-L^b^ was approximately 250% more potent than H-L^a^ against SK-OV-3 cells. Therefore, we further studied the inhibition of telomerase activity in SK-OV-3 cells by the two oxoisoaporphine ligands H-L^a^ (100 µM) and H-L^b^ (IC_50_ = 39.5 µM) through TRAP-silver staining assay.

### 2.3. Telomerase Activity Inhibition Studies by TRAP-Silver Staining Assay

**Figure 4:**
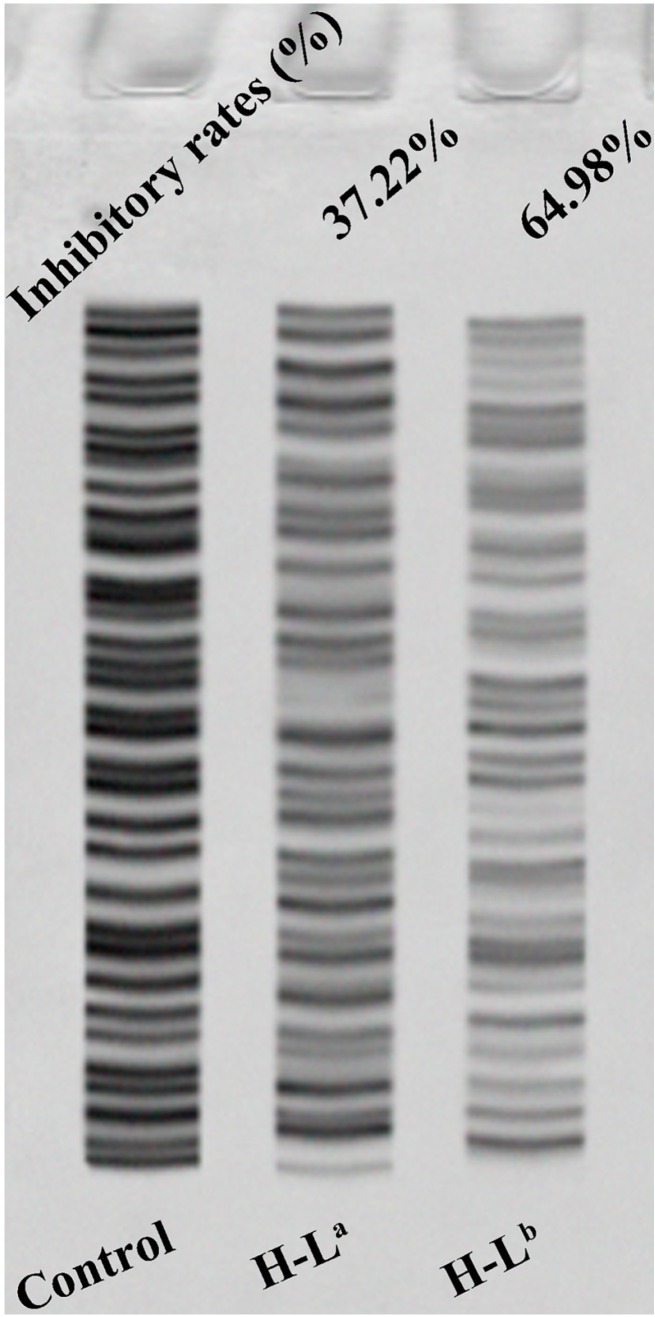
The inhibition of telomerase in SK-OV-3 cells by oxoisoaporphine ligands.

Recent studies found that about 80%–90% of various cancer cells have detectable telomerase activity; telomerase is therefore believed to be a potential anticancer target [27,28]. The results of the MTT assay and molecular docking suggested that it was of interest to compare to what extent the two oxoisoaporphine ligands H-L^a^ and H-L^b^ could inhibit telomerase activity in SK-OV-3 cells. This comparison was made by running the telomerase activity inhibition studies by the TRAP-silver staining assay with the two oxoisoaporphine ligands. As shown in Figure 4, the number of bands clearly decreased with each compound, showing that the telomerase activity was reduced by 64.98% when 39.5 μM H-L^b^ was applied, whereas the reduction was only 37.22% when 100 µM H-L^a^ was applied. This result was in good agreement with the results of MTT assay and molecular docking.

## 3. Experimental Methods

### 3.1. Materials and Instrumentation

The human normal liver cell line HL-7702 and the human cancer cell lines HCT-8, T-24, SK-OV-3/DDP, Hep-G2, SK-OV-3, BEL-7402, and NCI-H460 were obtained from the Institute of Biochemistry and Cell Biology, Chinese Academy of Sciences (Shanghai, China). The two test compounds were stored as stock solutions (2.0 mM) in DMSO, and further diluted with appropriate buffer to working concentrations (e.g., 100 µM of H-L^a^ and 39.5 µM of H-L^b^, respectively) when needed. MTT assay was performed on an M1000 microplate reader (Tecan Trading Co. Ltd., Shanghai, China).

### 3.2. Synthesis of Oxoisoaporphine Ligands

The two oxoisoaporphine ligands H-L^a^ and H-L^b^ were prepared according to the procedures previously reported by Chen and co-workers [19,21].

### 3.3. MTT Assay and Telomerase Activity Assay

The MTT assay and the telomerase activity assay by TRAP-silver staining assay followed the regular settings reported in previous work [20,21,29,30].

### 3.4. Docking Study

A docking study was performed using the AutoDock software (Version: 4.2.6, The Scripps Research Institute) in combination with MGLTools (Version: 1.5.6, The Scripps Research Institute) and PyMOL (Version: 1.7, Schrödinger Inc., New York, NY, USA) to explore the interactions between the ligands (H-L^a^ and H-L^b^) and hTR (PDB ID: 2INA), and to visualize the probable binding mode. All calculations were carried out on a Linux station (4 × 2 cores) running Centos 7. The three-dimensional structures of the two ligands were constructed using ChemBio3D Ultra 12.0 (Cambridge Soft Corporation, Cambridge, MA, USA) and then energetically minimized. They were then molecularly modeled using AutoDock. The 3D structure model of hTR can be obtained by the conversion of the data downloaded from the Protein Data Bank (http://www.rcsb.org, PDB ID: 2INA) using PyMOL, and the resulting image is shown in Figure 2A. Prior to molecular modeling, polar hydrogen atoms were added [22,23,24,25,26,31,32]. In the docking model, a grid (100 × 100 × 100 Å) was set, and the binding energies were obtained by AutoDock scoring function. After the complex of the ligand bound to the receptor was obtained, the following analyses were carried out using PyMOL software (Version: 1.7, Schrödinger Inc., New York, NY, USA).

## 4. Conclusions

The binding affinity of two oxoisoaporphine ligands toward telomerase was evaluated by molecular docking. The ligand H-L^a^ could form two hydrogen bonds with telomerase in the complex, whereas the ligand H-L^b^ could form four hydrogen bonds in the complex. The calculated binding energies of H-L^a^ and H-L^b^ with hTR were −6.43 and −9.76 kcal/mol, respectively. To examine the abilities of two oxoisoaporphine ligands to bind strongly to the telomerase inhibitory activity and to inhibit cancer proliferation, we also performed a TRAP assay and in vitro cytotoxicity studies of ligands H-L^a^ and H-L^b^. The results indicated that H-L^b^ was a potent inhibitor of telomerase and SK-OV-3 cell proliferation, and might be a potential lead compound for the development of a new telomerase inhibitor.
